# Dihydroartemisinin inhibits the expression of von Willebrand factor by downregulation of transcription factor ERG in endothelial cells

**DOI:** 10.1111/fcp.12622

**Published:** 2020-11-07

**Authors:** Fengyun Dong, Xinghai Zhao, Jianning Wang, Xin Huang, Xiao Li, Liang Zhang, Haixin Dong, Fuhong Liu, Mengge Fan

**Affiliations:** ^1^ Department of Laboratory Medicine Affiliated Hospital of Jining Medical University, Jining Medical University 89 Guhuai Road Jining Shandong 272029 China; ^2^ Shandong Provincial Qianfoshan Hospital, The First Affiliated Hospital of Shandong First Medical University 16766 Jingshi Road Jinan Shandong 250014 China; ^3^ Department of Urology Shandong Provincial Qianfoshan Hospital, The First Affiliated Hospital of Shandong First Medical University 16766 Jingshi Road Jinan Shandong 250014 China; ^4^ Department of Clinical Pharmacy Shandong Provincial Qianfoshan Hospital, The First Affiliated Hospital of Shandong First Medical University 16766 Jingshi Road Jinan Shandong 250014 China; ^5^ College of Traditional Chinese Medicine Shandong University of Traditional Chinese Medicine 16369 Jingshi Road Jinan Shandong 250011 China; ^6^ Shandong Provincial Key Laboratory of Animal Resistance Biology Institute of Biomedical Sciences College of Life Sciences Shandong Normal University 88 Wenhuadong Street Jinan Shandong 250014 China; ^7^ Laboratory of Microvascular Medicine Medical Research Center Shandong Provincial Qianfoshan Hospital, The First Affiliated Hospital of Shandong First Medical University 16766 Jingshi Road Jinan Shandong 250014 China; ^8^ Graduate School Shandong First Medical University & Shandong Academy of Medical Sciences 6699 Qingdao Road Jinan 250000 China

**Keywords:** dihydroartemisinin, ERG, human umbilical vein endothelial cells, von Willebrand Factor

## Abstract

Dihydroartemisinin (DHA), a semi‐synthetic derivative of artemisinin, has effective antitumor and anti‐inflammatory actions. von Willebrand factor (vWF), a large multifunctional glycoprotein, has a prominent function in hemostasis and is a key factor in thrombus formation. In addition, vWF has been regarded as a prospective biomarker for the diagnosis of endothelial dysfunction. In our experiment, we observed that 25 μM DHA specifically downregulated the expression of vWF mRNA and protein in human umbilical vein endothelial cells (HUVECs). Further investigations demonstrated that this DHA‐decreased vWF expression was mediated by the transcription factor ERG and not GATA3. Luciferase activity assay confirmed that DHA regulated the ERG binding with the −56 ETS‐binding motif on the human vWF promoter. Thus, the −56 ETS motif on the vWF promoter region regulates the expression of vWF gene which is induced by DHA. Taken together, we proved that DHA decreased the vWF transcription through the downregulation of ERG in HUVECs. As vWF plays a key role in vascular homeostasis, our findings suggest a new role of DHA in vascular diseases.

**Abbreviations**
DHA: Dihydroartemisinin
HUVECs: human umbilical vein endothelial cells
vWF: Von Willebrand factor
VEGF: vascular endothelial growth factor
ECs: endothelial cells

## Introduction

Artemisinins are extracted from sweet wormwood (*Artemisia annua*) and are extensively used to treat multidrug‐resistant malaria [1]. Dihydroartemisinin (DHA), a semi‐synthetic derivative of artemisinin, has greater effects as an antimalarial agent than artemisinin, is more water‐soluble, and has fewer adverse side effects [2]. Artemisinin and its derivatives, including DHA, have also significant antitumor and anti‐inflammatory activities in vitro and in vivo [3, 4]. In cancer cells, DHA reduces the expression of vascular endothelial growth factor (VEGF) and decreases the binding of VEGF to its receptor [5]. In endothelial cells (ECs), DHA reduces the cell proliferation, migration, and tube formation [6, 7, 8]. Moreover, DHA also regulates the expression of VEGFR1, VEGFR2, VE‐cadherin, and occludin in ECs [9, 10, 11, 12]. However, the role of DHA on the expression of von Willebrand factor (vWF) in ECs has not been described.

The vWF is produced by ECs, platelets, and megakaryocytes. It is a large multimeric glycoprotein that mediates platelet hemostatic function [13]. The vWF in circulation is essentially derived from the ECs [13]; it is synthesized in the ECs and stored in Weibel–Palade bodies [14, 15]. When endothelium is damaged, vWF enables the capture of the platelets at the damaged sites [16, 17, 18]. Recent reports have implicated that as the plasma vWF levels increase, the risk of coronary heart disease increases as well [19, 20]; consequently, vWF is known as an independent predictor of cardiovascular diseases [21]. In addition, prospective studies have described that the occurrence of acute ischemic stroke (AIS) is also associated with high levels of vWF [22]. Moreover, vWF is also regarded as a promising biomarker for endothelial dysfunction [23, 24].

vWF expression is regulated by genetic, physiological, and environmental factors. Inflammation and hypoxic stimulation may increase vWF secretion [25, 26]. Further, a low dose of cadmium can promote the vWF expression in ECs [27]. The vWF promoter includes several regulatory regions and specific transcription factors regulate them [28, 29]. It has been recently shown that the ETS factor ERG interacts with endothelial‐specific genes, including vWF [30]. Besides, GATA3 is also a positive transcription factor regulating the expression of vWF [31, 32]. Thus, the ETS and GATA factors are possibly responsible for regulating the vWF expression by mediating the response to extracellular stimuli.

In our study, we explored the influence of DHA on vWF expression in vitro. We believe that our study provides a theoretical basis that indicates DHA as a therapeutic agent for vascular‐related diseases associated with elevated vWF.

## Materials and methods

### Cell culture and treatments

HUVECs were obtained from Lonza (Basel, Switzerland) and cultured in EBM‐2 basal endothelium medium (serum free) with EGM‐2 SingleQuots (Lonza, Walkersville, MD, USA) and penicillin/streptomycin solution. DHA was obtained from Sigma‐Aldrich (St. Louise, MO, USA) and was dissolved in dimethylsulfoxide (DMSO). HUVECs were incubated with DHA at a final concentration of 25 μM for 24 h before further use. DMSO alone was used as the control in all experiments.

### RNA extraction and quantitative real‐time PCR (qRT‐PCR)

The treated HUVECs were collected, and the total cellular RNA was isolated with E.Z.N.A.^®^ Total RNA Kit II (OMEGA, Norcross, GA, USA) following the manufacturer’s protocol. The RevertAid First Strand cDNA Synthesis Kit (Thermo Fisher Scientific, Grand Island, NY, USA) was used to synthesize the cDNA. qRT‐PCR was conducted with a CFX96 Real‐Time System (Bio‐Rad, Hercules, California, USA). Each PCR was repeated in triplicates. The primer sequences are shown in *Table* *1*.

**Table 1:** qRT‐PCR primer sequences

Gene	Sequence	Size (bp)	Tm (°C)
vWF			
Forward	CGGCTTGCACCATTCAGCTA	90	61.5
Reverse	TGCAGAAGTGAGTATCACAGCCATC		
GATA3		92	60.95
Forward	GTTGCACAGGTAGTGTCCCG		
Reverse	GGTCCAGCACAGAAGGCA		
ERG			
Forward	AGTGAGGACCAGTCGTTG	151	56.18
Reverse	TAGGGTTACATTCCATTTTG		
GAPDH			
Forward	TGATGACATCAAGAAGGTGGTGAAG	240	60
Reverse	TCCTTGGAGGCCATGTGGGCCAT		

Note:

All sequences are in the 5' to 3' orientation. Tm, melting temperature.

### Western blot analysis

Western blot was performed as previously described [27]. Primary antibodies used were rabbit anti‐vWF (1:1000, A008229) antibodies obtained from Dako (Glostrup, Denmark), and rabbit anti‐ERG (1:3000, ab133264) and rabbit anti‐GATA3 (1:1000, ab199428) antibodies obtained from Abcam (Cambridge, MA, USA). Rabbit anti‐GAPDH (1:8000, 2118) antibodies were obtained from Cell Signaling Technology (Danvers, MA, USA). A horseradish‐peroxidase (HRP)‐linked goat anti‐rabbit IgG was used as the secondary antibody. The immunoreactive bands were detected using ECL reagents (Millipore, Billerica, MA, USA), and the densitometric analysis of Western blots was performed using ImageJ software (NIH, Bethesda, MD, USA).

### Immunofluorescence

HUVECs were grown on an adhesive culture dish (Sigma‐Aldrich) and then treated with 25 μM DHA. After 24 h of stimulation, the cells were washed and fixed for 10 min with 4 % paraformaldehyde and then blocked with 5 % bovine serum albumin. Next, the samples were incubated with a rabbit polyclonal antibody anti‐human vWF (1:200, A008229, Dako) for one night at 4 °C and incubated with an Alexa Fluor 546 secondary antibody (A10040, Life Technologies) for 2 h at normal temperature. The nuclei were stained with DAPI (Roche; Mannheim, Germany). The samples were pictured with an Olympus FSX100 Imaging System (Olympus Corporation, Tokyo, Japan), and the excitation wavelength was 546 nm.

### Plasmid transfection

Overexpression plasmids for human ERG (RG208093), GATA3 (SC108486), and control plasmids (empty vector, EV) were purchased from OMEGA (Norcross, GA, USA). The cell transfections were implemented with Lipofectamine 3000 (Thermo Fisher Scientific) following the manufacturer’s protocol. Briefly, the HUVECs were cultured in 6‐well plates and transfected with 2000 ng plasmids when the cells reached 70–80 % confluency. After transfection for 24 h, the cells were incubated in DHA at a final concentration of 25 uM for the next 24 h. The overexpression of ERG and GATA3 was detected by qRT‐PCR and Western blot analysis.

### Luciferase reporter assay

The −487 to +246 fragment of the vWF promoter was generated by DNA synthesis. The −56 ERG mutation (5′‐TTTCCTT‐3′ to 5′‐TTTAATT‐3′) and +220 GATA3 mutation (5′‐GATA‐3′ to 5′‐AACA‐3′) on the vWF promoter fragment were cloned into a pGL3‐basic vector (Promega, Madison, WT) produced by PCR. The resulting pGL3‐vWF (wild‐type, WT) and pGL3‐vWF (mutant, mut) constructs were transfected into the HUVECs with Lipofectamine 3000 (Thermo Fisher Scientific) following the manufacturer’s protocols. A plasmid containing the Renilla luciferase reporter gene was used as an internal control. After 48 h, 25 μM DHA was added to the culture and the cells were collected by passive lysis buffer after a 24‐h treatment. The cell lysates were examined using a dual‐luciferase reporter assay system (Promega) and measured by a GloMax‐Multi Jr Single Tube Multimode Reader (Promega) at wavelengths of 350–650 nm.

### Statistical analysis

All data were presented as mean ± SEM. The difference between two groups was estimated with Student’s *t*‐test (two‐tailed), which was performed in SPSS 18.0 and GraphPad Prism 5 software. *P* value < 0.05 was regarded as significant.

## Results

### DHA decreases the expression of vWF in HUVECs

**Figure 1:**
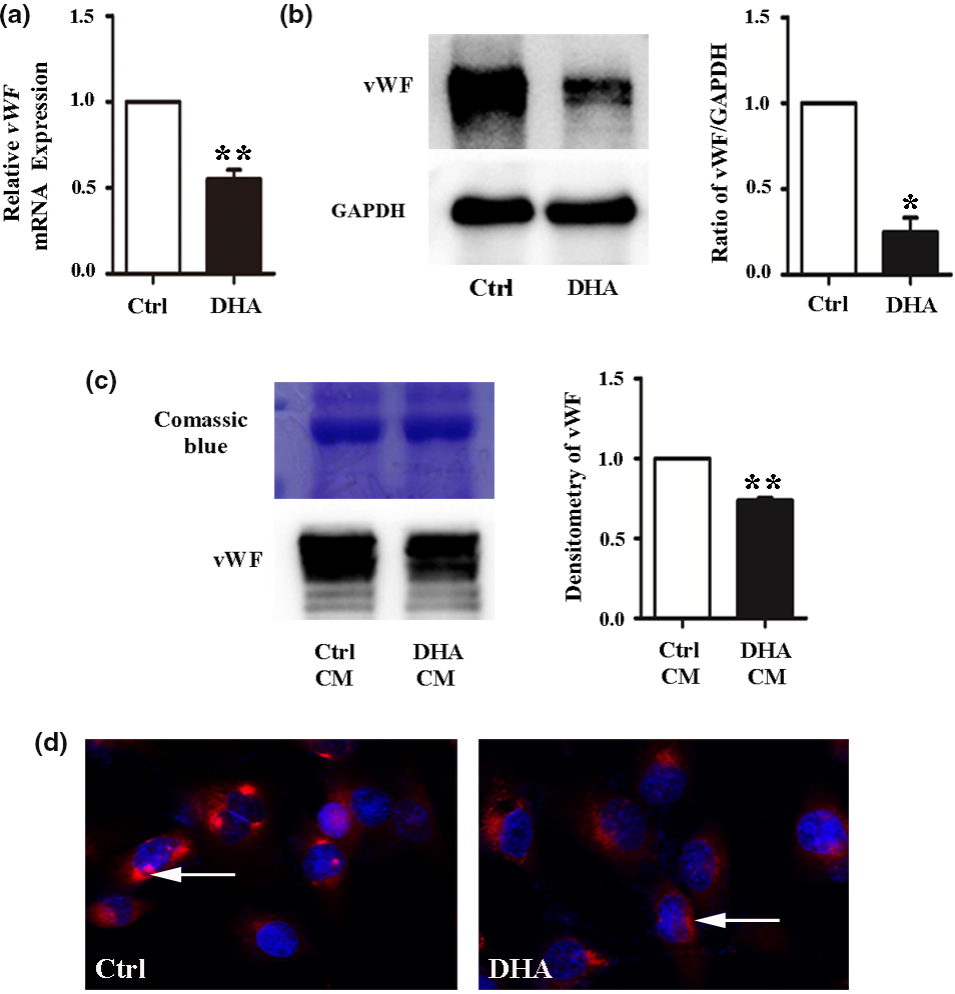
DHA suppresses vWF expression in HUVECs. (a) Relative vWF mRNA expression measured by quantitative real‐time PCR(qRT‐PCR) in human umbilical vein endothelial cells (HUVECs) treated with 25 μM DHA. *n* = 4; **, *P* < 0.01. (b) Representative immunoblots and densitometric analysis of vWF and GAPDH in 25 μM DHA‐treated HUVECs. *n* = 3; *, *P* < 0.05. (c) Representative immunoblots and densitometric analysis of vWF in HUVECs with control or DHA conditioned medium for 24 h. *n* = 3; **, *P* < 0.01. Coomassie blue staining of the gel was used as the loading control. (d) Immunofluorescent staining of vWF in HUVEC monolayers treated with dimethylsulfoxide (DMSO) (control) and 25 μM DHA for 24 h. The arrow refers to vWF staining in the cells

HUVECs were treated with 25 µM DHA for 24 h. The results of qRT‐PCR and Western blotting showed that DHA significantly decreased the mRNA (*Figure* *1a*) and protein levels (*Figure* *1b*) of vWF. In addition, Western blotting indicated that the extracellular vWF from the culture media was decreased after DHA treatment (*Figure* *1c*). Consistent with the above results, we observed that after immunofluorescence, the vWF expression on the ECs membrane and cytoplasm was distinctly decreased after DHA treatment (*Figure* *1d*). Thus, our results show that DHA decreases the expression of vWF in HUVECs.

### DHA downregulates ERG and GATA3 expression in HUVECs

**Figure 2:**
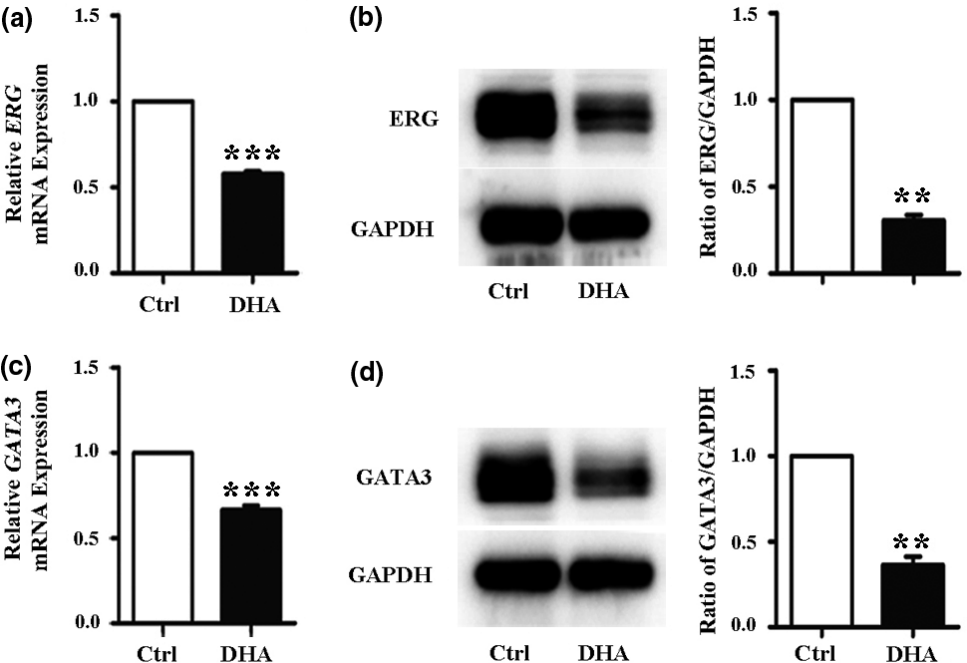
DHA decreases ERG and GATA3 expression in HUVECs. (a) Relative ERG mRNA expression measured by quantitative real‐time PCR (qRT‐PCR) in human umbilical vein endothelial cells (HUVECs) treated with 25 μM DHA. *n* = 4; ***, *P* < 0.001. (b) Representative immunoblots and densitometric analysis of ERG and GAPDH in HUVECs treated with 25 μM DHA. *n* = 3; **, *P* < 0.01. (c) Relative GATA3 mRNA expression measured by qRT‐PCR in HUVECs treated with 25 μM DHA. *n* = 4; ***, *P* < 0.001. (d) Representative immunoblots and densitometric analysis of GATA3 and GAPDH in HUVECs treated with 25 μM DHA. *n* = 3; **, *P* < 0.01

Transcription factors GATA3 and ERG are major regulators of vWF [33]. We investigated the effect of DHA on GATA3 and ERG expression. HUVECs were incubated with 25 μM DHA for 24 h, and the protein and mRNA levels of GATA3 and ERG were examined. The qRT‐PCR results showed that ERG and GATA3 mRNA expression levels were remarkably decreased after DHA treatment (*Figure* *2a* *and* *c*). The results of Western blot analysis showed a similar pattern of decrease of GATA3 and ERG protein levels (*Figure* *2b* *and* *d*). These results indicate that the downregulation of ERG and GATA3 may be a cause for the decreased expression of vWF induced by DHA in HUVECs.

### ERG mediates the DHA‐induced downregulation of vWF and not GATA3

**Figure 3:**
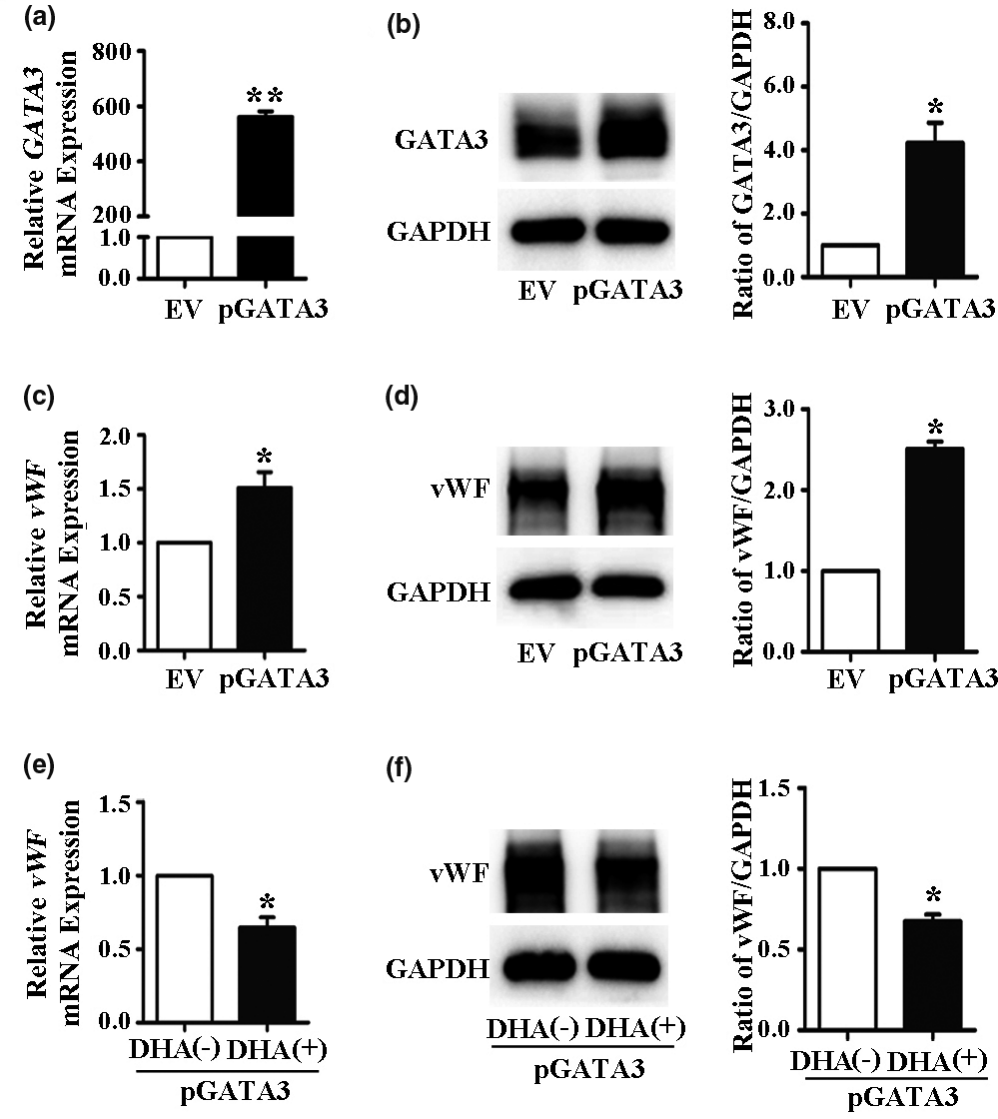
Overexpression of GATA3 does not eliminate the DHA‐mediated downregulation of vWF expression. (a) Relative GATA3 mRNA expression in human umbilical vein endothelial cells (HUVECs) transfected with empty vector (EV) or GATA3 plasmids as measured by quantitative real‐time PCR. *n* = 3; **, *P* < 0.01. (b) Representative immunoblots and densitometric analysis of GATA3 and GAPDH in HUVECs transfected with EV or GATA3 plasmids. *n* = 3; *, *P* < 0.05. (c) Relative vWF mRNA expression in HUVECs transfected with GATA3 plasmids. *n* = 4; *, *P* < 0.05. (d) Representative immunoblots and densitometric analysis of vWF and GAPDH in HUVECs transfected with GATA3 plasmids. *n* = 3; *, *P* < 0.05. (e) Relative vWF mRNA expression in HUVECs transfected with GATA3 plasmids and treated with 25 μM DHA for 24 h. *n* = 4; *, *P* < 0.05. (f) Representative immunoblots and densitometric analysis of vWF and GAPDH in HUVECs transfected with GATA3 plasmids and treated with 25 μM DHA for 24 h. *n* = 3; *, *P* < 0.05

**Figure 4:**
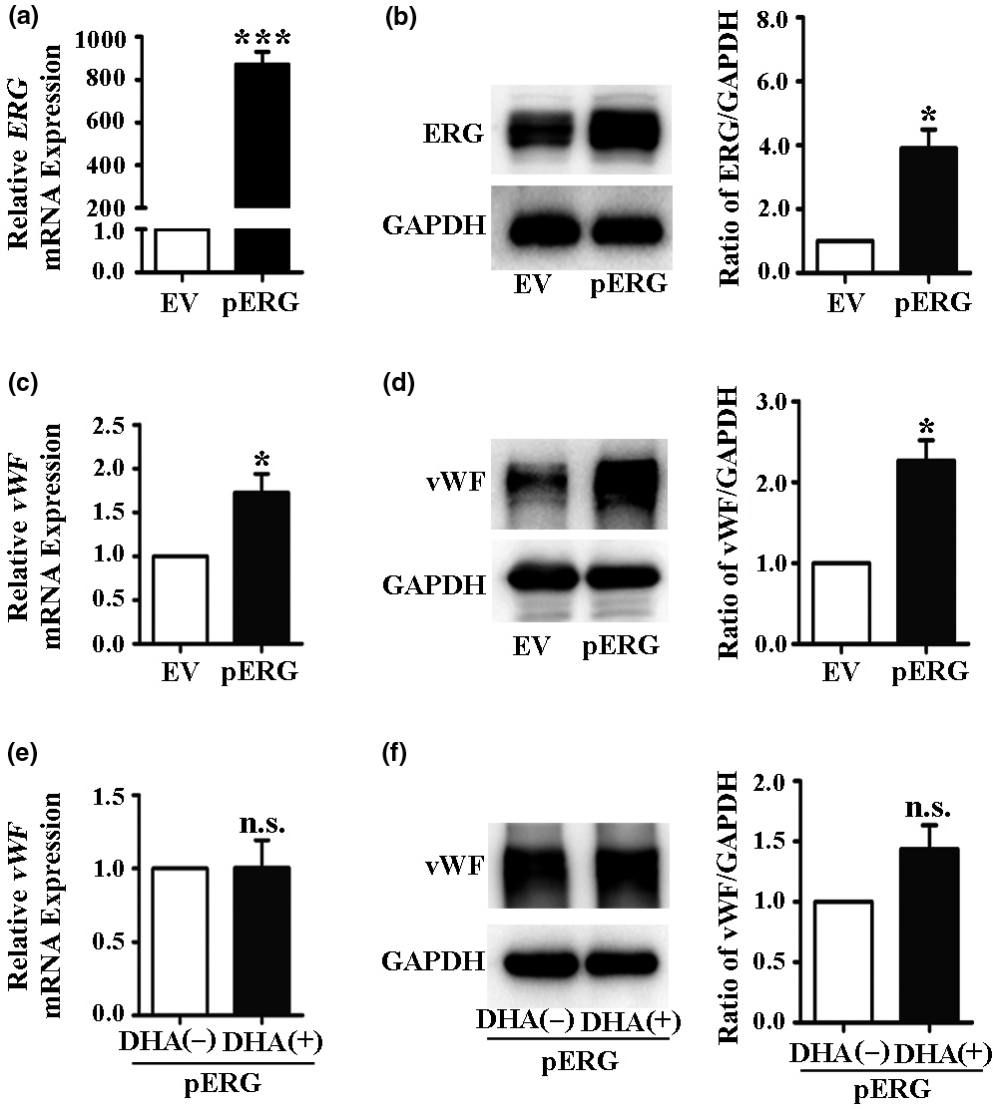
Overexpression of ERG eliminates DHA‐mediated downregulation of vWF expression. (a) Relative ERG mRNA expression in human umbilical vein endothelial cells (HUVECs) transfected with empty vector (EV) or ERG plasmids as measured by quantitative real‐time PCR. *n* = 4; ***, *P* < 0.001. (b) Representative immunoblots and densitometric analysis of ERG and GAPDH in HUVECs transfected with EV or ERG plasmids. *n* = 3; *, *P* < 0.05. (c) Relative vWF mRNA expression in HUVECs transfected with ERG plasmids. *n* = 4; *, *P* < 0.05. (d) Representative immunoblots and densitometric analysis of vWF and GAPDH in HUVECs transfected with ERG plasmids. *n* = 3; *, *P* < 0.05. (e) Relative vWF mRNA expression in HUVECs transfected with ERG plasmids and treated with 25 μM DHA for 24 h. *n* = 4; n.s., nonsignificant. (f) Representative immunoblots and densitometric analysis of vWF and GAPDH in HUVECs transfected with ERG plasmids and treated with 25 μM DHA for 24 h. *n* = 3; n.s., nonsignificant

To confirm the effects of GATA3 and ERG in the DHA‐mediated vWF expression, we transfected the HUVECs with ERG and GATA3 plasmids. The Western blot and qRT‐PCR results revealed that the protein and mRNA expression levels of ERG and GATA3 were remarkably increased by plasmid transfection (*Figure* *3a* *and* *b*, *Figure* *4a* *and* *b*). The expression of vWF in HUVECs was significantly increased after transfection with the GATA3 or ERG plasmids (*Figure* *3c* *and* *d*, *Figure* *4c* *and* *d*). Subsequently, the HUVECs were transfected with GATA3 or ERG plasmids and then treated with or without 25 μM DHA for 24 h to detect the expression of vWF. The Western blot and qRT‐PCR results also demonstrated that the DHA‐induced decrease in vWF expression was inhibited by ERG overexpression (*Figure* *4e* *and* *f*). However, DHA‐induced decrease in vWF expression was not blocked by GATA3 overexpression (*Figure* *3e* *and* *f*).

### ERG regulates vWF transcription through the −56 ETS‐binding elements

**Figure 5:**
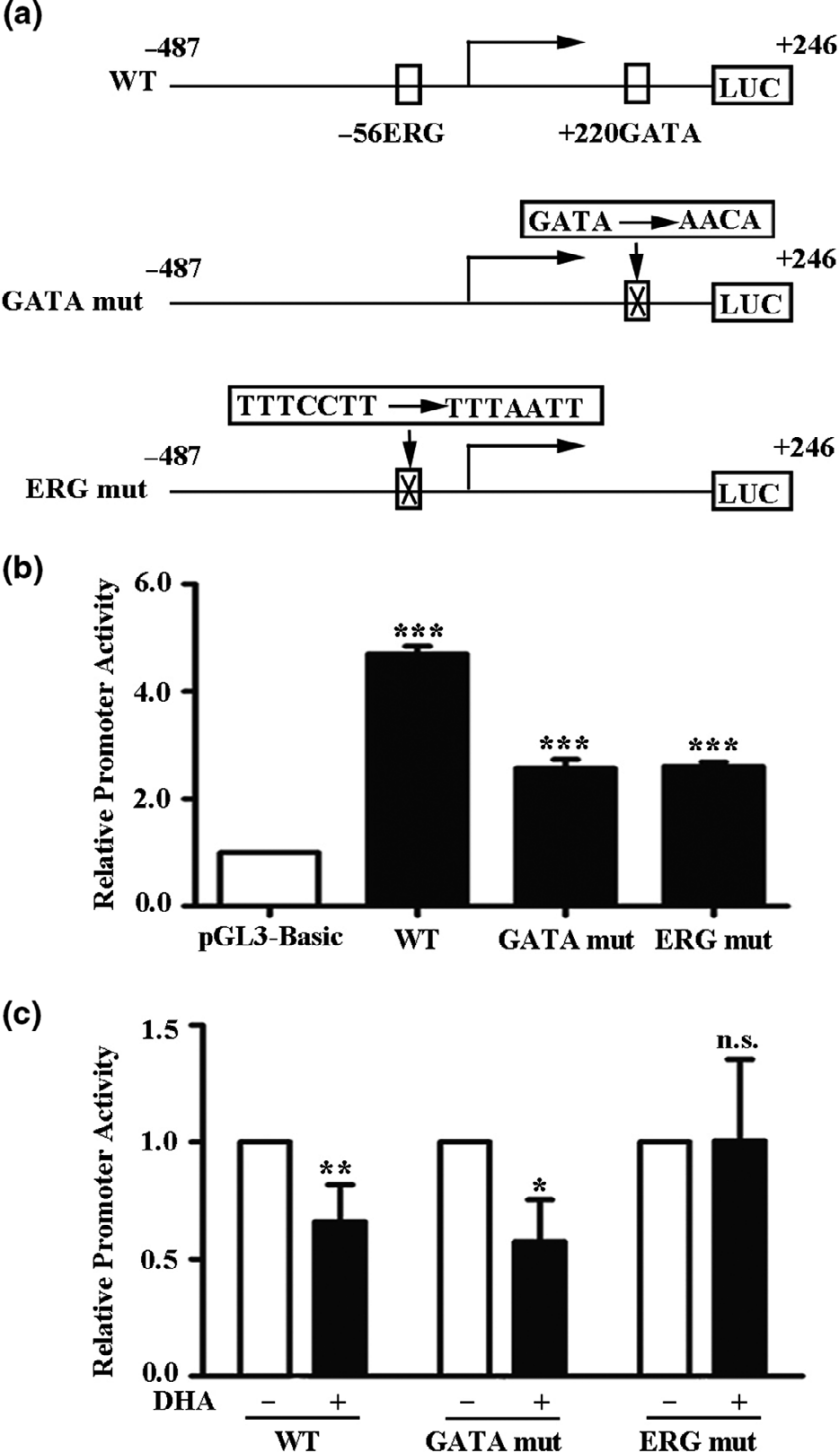
ERG regulates vWF transcription through the −56 ETS‐binding elements. (a) The schematic representation of luciferase construct of vWF promoter with or without a mutation of the −56 ERG motif or +220 GATA3 motif. (b) Luciferase assay of human umbilical vein endothelial cells (HUVECs) transiently transfected with pGL3‐basic, pGL3‐vWF (WT), or pGL3‐vWF containing a mutation of the +220 GATA3 motif (GATA mut) or a mutation of the −56 ERG motif (ERG mut). (c) Luciferase assay of human umbilical vein endothelial cells (HUVECs) treated with 25 μM DHA for 24 h and then transiently transfected with pGL3‐vWF (WT) or pGL3‐vWF containing a mutation of the +220 GATA3 motif or a mutation of the −56 ERG motif. The results show the means and standard deviations of the normalized luciferase light units. The mean of each column was compared to the mean of the control, and Student’s *t*‐test was used to determine the significance. *n* = 3; ***; *P* < 0.001. **; *P* < 0.01.*; *P* < 0.05; n.s., nonsignificant

ERG factors can bind to an ERG‐binding element (−56 nt), and the GATA3 factors can bind to a GATA‐binding element (+220 nt) on the promoter of vWF [31, 33]. To further clarify the mechanism of vWF downregulation by DHA, we generated the luciferase constructs of the vWF promoter (WT) with or without a mutation of the −56 ERG (ERG mut)‐ and +220 GATA3 (GATA3 mut)‐binding sites (*Figure* *5a*) and transfected them into HUVECs. In the absence of DHA, the vWF promoter (WT) activity was significantly upregulated compared with the activity in pGL3‐basic (*Figure* *5b*). The vWF mutant promoters (GATA3 mut or ERG mut) did not completely reduce the promoter activity to the level of the control (*Figure* *5b*). After DHA treatment, the vWF promoter (WT) activity was significantly downregulated (*Figure* *5c*). Further, the activity of the GATA‐binding site mutation promoter (GATA3 mut) was also downregulated by DHA treatment. However, the activity of the ERG‐binding site mutation promoter (ERG mut) was not affected by DHA treatment (*Figure* *5c*). These results suggest that the −56 ETS‐binding elements mediate DHA‐induced vWF transcription.

## Discussion

Dihydroartemisinin is an effective antimalarial drug with few adverse effects and highly efficient antitumor and anti‐inflammatory effects [3, 4]. In our study, we discovered that the vWF mRNA and protein expression levels were downregulated by DHA in ECs. Moreover, we also found that the transcriptional factor ERG mediates this DHA‐induced vWF expression. These findings suggest that DHA treatment may counter vascular‐related diseases by downregulating vWF expression in ECs. Our research revealed a new mechanism through which DHA effects ECs; thus, this provides new evidence of the use of DHA as a therapeutic agent against vascular diseases associated with elevated vWF levels.

vWF regulates hemostasis and thrombosis, and has multiple roles in vascular inflammation and angiogenesis. Studies have shown that an elevated plasma vWF level is related to several vascular diseases, including coronary heart disease [19], atrial fibrillation [34], and hypertension [35]. In addition, vWF plays an important role in thrombus formation. Further, there is also a correlation between the vWF levels and stroke subtypes as well as the functional outcomes in AIS patients [36]. Therefore, suppressing the expression of vWF may reduce the incidence of the vascular diseases associated with elevated vWF levels.

Various genetic, physiological, and environmental elements regulate vWF expression. Our previous research has confirmed that a low‐dose Cd exposure is associated with the increased secretion of vWF in ECs [27]. In the present study, we showed that DHA downregulated the level of vWF in ECs. This implies that the roles that DHA plays in the vascular system might be affected by vWF, and thereby this may suggest a novel role of DHA in clinical treatment. Previous studies have confirmed that the cellular response of DHA toward ECs includes cell autophagy, proliferation, migration, and tube formation [37, 38]. In the present study, the results showed that DHA inhibits the expression of vWF, which has not been reported in previous studies.

To explore the potential molecular mechanisms of DHA‐induced downregulation of vWF, we examined the expression GATA3 and ERG transcription factors in this study. The GATA family members are key transcriptional regulators [39]. These factors share a similar DNA‐binding domain, in the gene promoter to directly activate or inhibit the expression of the target genes [40]. GATA3 is abundantly expressed in ECs and showed a significant binding affinity on the vWF promoter [31, 32]. In the present study, GATA3 was markedly decreased by DHA; however, overexpression of GATA3 did not diminish DHA‐induced vWF downregulation. This indicated that GATA3 does not mediate the downregulation of vWF expression induced by DHA. Other GATA family members, such as GATA2 and GATA6, are also bound to the vWF promoter [31] and may exert their functions. However, the activity of the vWF promoter with GATA‐binding site mutation (+220 nt) was still downregulated by DHA treatment suggesting that the +220 GATA motif does not mediate DHA regulated vWF transcription. Therefore, GATA family, including GATA3, may not participate in the repression of vWF expression induced by DHA.

The transcription factor ERG is a member of the ETS transcription factor family and is expressed at high levels in ECs [41]. ERG is involved in vascular homeostasis and is a transcriptional activator for several EC‐specific genes such as endoglin, vWF, VE‐cadherin, intercellular adhesion molecule, and claudin‐5 [42, 43, 44, 45, 46, 47]. Our results revealed that ERG and vWF were significantly decreased by DHA, and the overexpression of ERG by plasmids moderated the downregulation of vWF, which was induced by DHA. Furthermore, ERG binds to the −56 ETS site and this subsequent DNA–protein interaction positively mediates vWF expression [33]. This is consistent with our findings as the activity of the ERG‐binding site mutation promoter was unaffected by DHA treatment; hence, the −56 ETS‐binding elements may have mediated the DHA‐induced vWF downregulation of transcription.

In summary, we proved that DHA decreases the expression of vWF via downregulating the ERG transcription factor. This is a novel mechanism underlying the role of DHA in the endothelium, and we believe that this will help further the exploration of its clinical application.

## Conflicts of interest

The authors have no conflicts of interest to declare.
